# Supplementary material for: Global Cropland Connectivity: A Risk Factor for Invasion and Saturation by Emerging Pathogens and Pests
Source: Bioscience. 2020 Jul 29;70(9):744–58. doi: 10.1093/biosci/biaa067 (PMC7498352; doi:10.1093/biosci/biaa067)
Supplement: biaa067_Supplemental_Files [file biaa067_supplemental_files.zip › figure_S1.pdf]

Global Cropland Connectivity: A Risk Factor for Invasion and Saturation by Emerging Pathogens and Pests

Y. XING*, J. F. HERNANDEZ NOPSA*, K. F. ANDERSEN, J. ANDRADE-PIEDRA, F. D. BEED, G. BLOMME, M. CARVAJAL-YEPES, D. L. COYNE, W. J. CUELLAR, G. A. FORBES, J. F. KREUZE, J. KROSCHEL, P. L. KUMAR, J. P. LEGG, M. PARKER, E. SCHULTE-GELDERMANN, K. SHARMA, AND K. A. GARRETT

*These authors made equivalent contributions

Y. Xing, J. F. Hernandez Nopsa, K. F. Andersen, and K. A. Garrett (karengarrett@ufl.edu) are affiliated with the Plant Pathology Department, Institute for Sustainable Food Systems, and Emerging Pathogens Institute at University of Florida, Gainesville, USA. J. F. Hernandez Nopsa is affiliated with Corporación Colombiana de Investigación Agropecuaria, AGROSAVIA, Mosquera-Bogota, Colombia. J. Andrade-Piedra, G. A. Forbes, J. F. Kreuze, and J. Kroschel are affiliated with International Potato Center (CIP), P.O. Box 1558, Lima 12, Peru. F. D. Beed is affiliated with Plant Production and Protection Division, Food and Agriculture Organization of the United Nations (FAO), 00153 Roma, Italy. G. Blomme is affiliated with Bioversity International, c/o ILRI, Addis Ababa, Ethiopia. M. Carvajal-Yepes and W. J. Cuellar are affiliated with International Center for Tropical Agriculture (CIAT), AA6713, Cali, Colombia. D. L. Coyne is affiliated with International Institute of Tropical Agriculture (IITA), Nairobi, Kenya. P. L. Kumar is affiliated with International Institute of Tropical Agriculture (IITA), Ibadan, Nigeria. J. P. Legg is affiliated with International Institute of Tropical Agriculture (IITA), Dar es Salaam, Tanzania. M. Parker, E. Schulte-Geldermann, and K. Sharma are affiliated with

International Potato Center (CIP), Nairobi, Kenya. All authors are affiliated with the CGIAR Research Program on Roots, Tubers and Bananas (RTB).

This PDF file includes:

Figures S1

Supplemental Figures

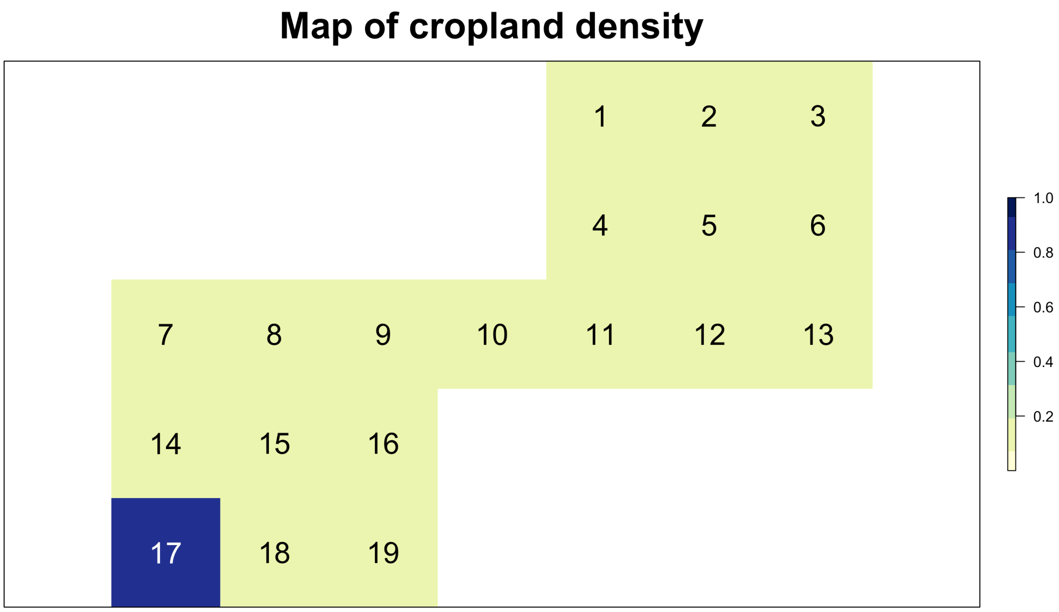

Figure S1a. The hypothetical landscape from Fig. 2 in the main text, used to illustrate how the cropland connectivity risk index (CCRI) functions. In the main text, an example for dispersal described by the negative exponential with parameter γ =0.7 and link threshold = 0.001 is given, and repeated here as Fig. S1d. In the following cases, additional examples are illustrated for other values of the negative exponential and for the inverse power law function. Fig. 2H-L in the main text includes uncertainty quantification to evaluate the responses across the four parameter combinations used in these illustrations.

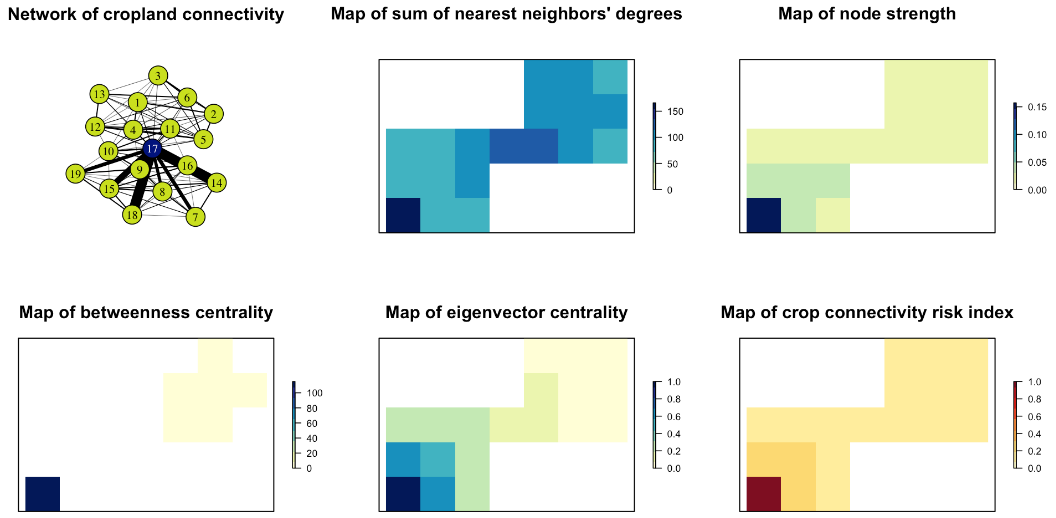

Figure S1b. For distribution probabilities following an inverse power law model with parameter β = 1.5 and with a link threshold level of 0.001, this figure shows the network based on the map from Figure S1a, the maps of the four node measures of which the cropland connectivity risk index (CCRI) is a function, and the resulting CCRI, itself

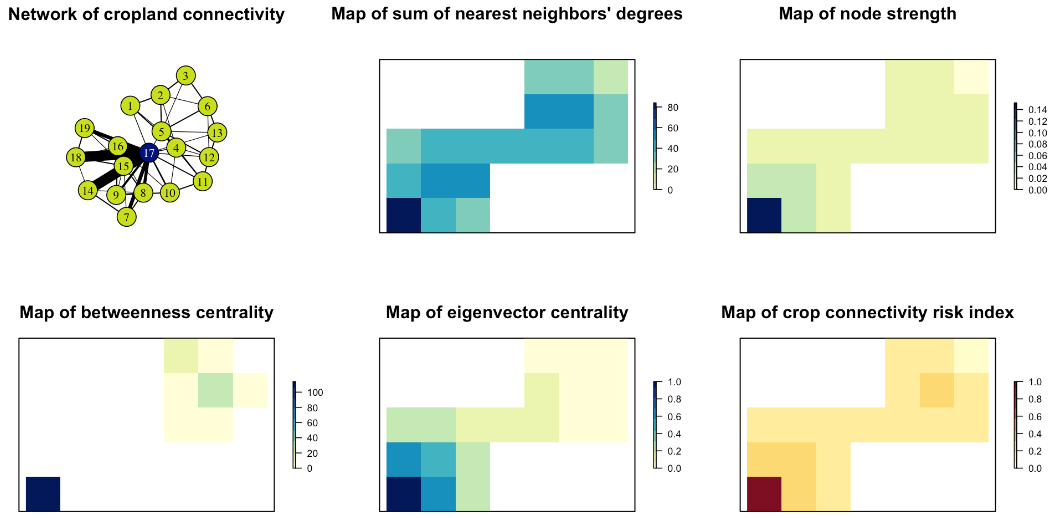

Figure S1c. For distribution probabilities following an inverse power law model with parameter β = 1.5 and with a link threshold level of 0.002, this figure shows the network based on the map from Figure S1a, the maps of the four node measures of which the cropland connectivity risk index (CCRI) is a function, and the resulting CCRI, itself

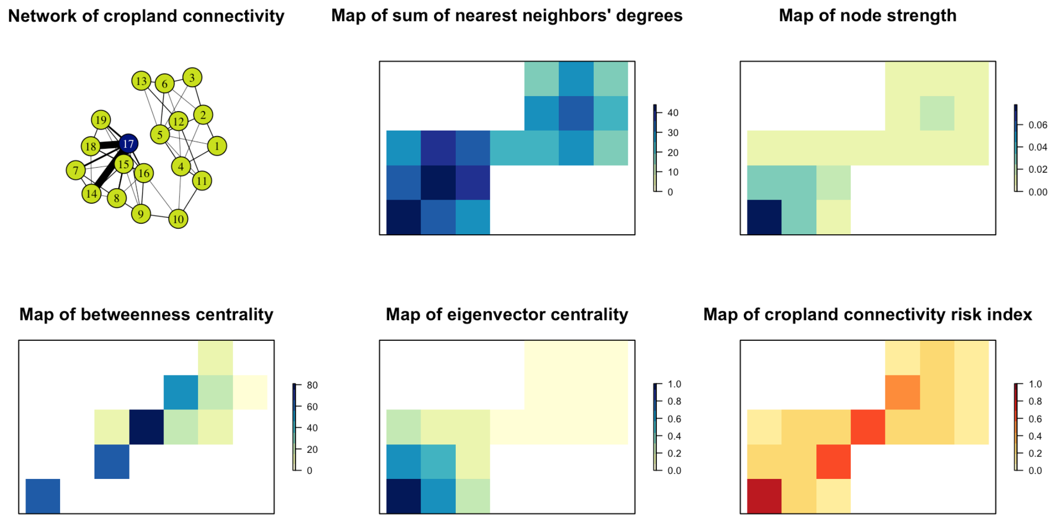

Figure S1d. For distribution probabilities following a negative exponential model with parameter $\gamma = 0.7$ and with a link threshold level of 0.001, this figure shows the network based on the map from Figure S1a, the maps of the four node measures of which the cropland connectivity risk index (CCRI) is a function, and the resulting CCRI, itself, [Note that this parameter combination is the same as in Fig. 2 in the main text]

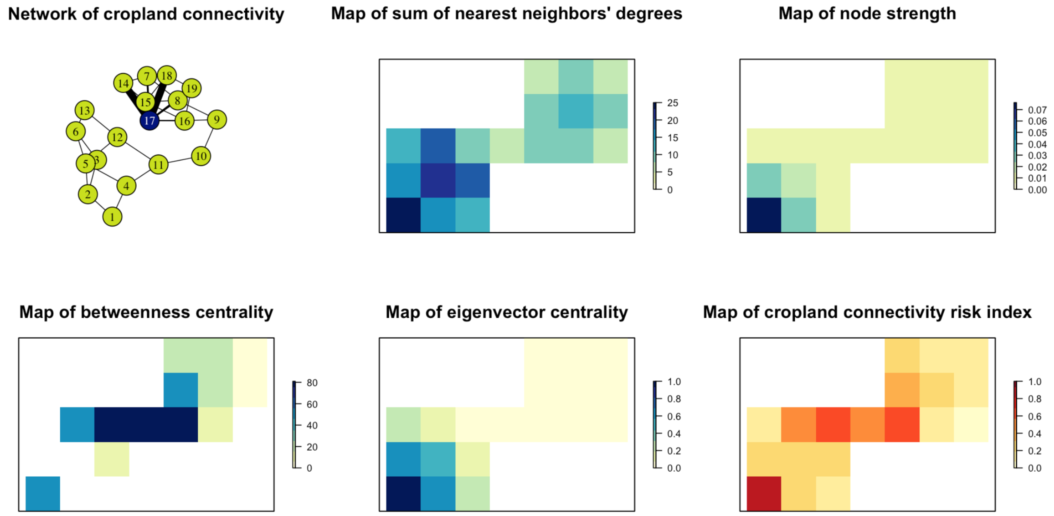

Figure S1e. For distribution probabilities following a negative exponential model with parameter $\gamma = 0.7$ and with a link threshold level of 0.002, this figure shows the network based on the map from Figure S1a, the maps of the four node measures of which the cropland connectivity risk index (CCRI) is a function, and the resulting CCRI, itself
