# Supplementary material for: Global Cropland Connectivity: A Risk Factor for Invasion and Saturation by Emerging Pathogens and Pests
Source: Bioscience. 2020 Jul 29;70(9):744–58. doi: 10.1093/biosci/biaa067 (PMC7498352; doi:10.1093/biosci/biaa067)
Supplement: biaa067_Supplemental_Files [file biaa067_supplemental_files.zip › figure_S4.pdf]

Global Cropland Connectivity: A Risk Factor for Invasion and Saturation by Emerging Pathogens and Pests

Y. XING*, J. F. HERNANDEZ NOPSA*, K. F. ANDERSEN, J. ANDRADE-PIEDRA, F. D. BEED, G. BLOMME, M. CARVAJAL-YEPES, D. L. COYNE, W. J. CUELLAR, G. A. FORBES, J. F. KREUZE, J. KROSCHEL, P. L. KUMAR, J. P. LEGG, M. PARKER, E. SCHULTE-GELDERMANN, K. SHARMA, AND K. A. GARRETT

*These authors made equivalent contributions

Y. Xing, J. F. Hernandez Nopsa, K. F. Andersen, and K. A. Garrett (karengarrett@ufl.edu) are affiliated with the Plant Pathology Department, Institute for Sustainable Food Systems, and Emerging Pathogens Institute at University of Florida, Gainesville, USA. J. F. Hernandez Nopsa is affiliated with Corporación Colombiana de Investigación Agropecuaria, AGROSAVIA, Mosquera-Bogota, Colombia. J. Andrade-Piedra, G. A. Forbes, J. F. Kreuze, and J. Kroschel are affiliated with International Potato Center (CIP), P.O. Box 1558, Lima 12, Peru. F. D. Beed is affiliated with Plant Production and Protection Division, Food and Agriculture Organization of the United Nations (FAO), 00153 Roma, Italy. G. Blomme is affiliated with Bioversity International, c/o ILRI, Addis Ababa, Ethiopia. M. Carvajal-Yepes and W. J. Cuellar are affiliated with International Center for Tropical Agriculture (CIAT), AA6713, Cali, Colombia. D. L. Coyne is affiliated with International Institute of Tropical Agriculture (IITA), Nairobi, Kenya. P. L. Kumar is affiliated with International Institute of Tropical Agriculture (IITA), Ibadan, Nigeria. J. P. Legg is affiliated with International Institute of Tropical Agriculture (IITA), Dar es Salaam, Tanzania. M. Parker, E. Schulte-Geldermann, and K. Sharma are affiliated with

International Potato Center (CIP), Nairobi, Kenya. All authors are affiliated with the CGIAR Research Program on Roots, Tubers and Bananas (RTB).

This PDF file includes:

Figures S4

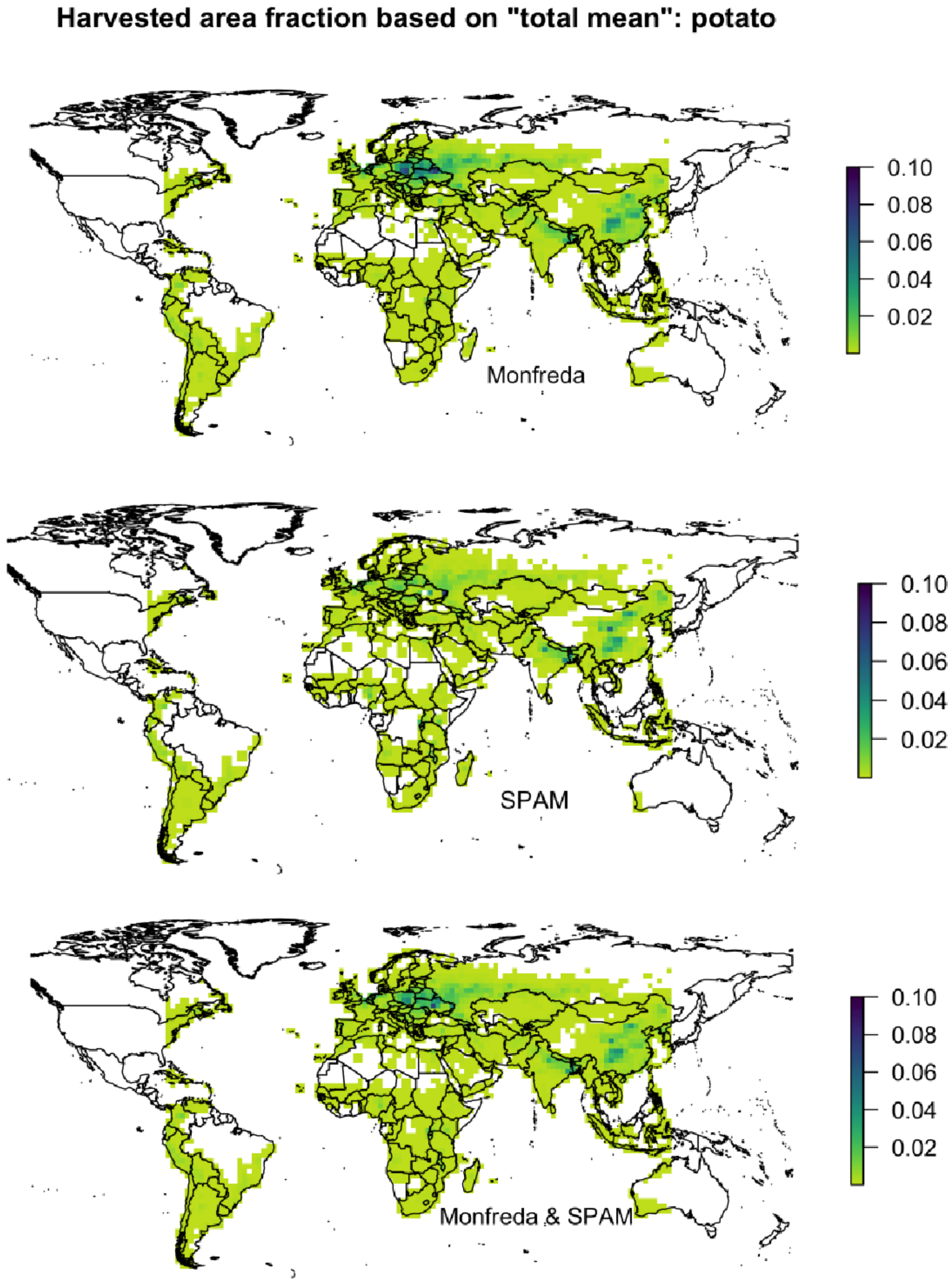

Figure S4a1. Maps of the harvested area fraction for **potato** based on the "**total mean**", the mean of all units within the area being aggregated (2-degree resolution, aggregated from 5-minute resolution in Monfreda et al. 2008, MapSPAM2005v3.2, and "Monfreda & SPAM" (the mean of the harvested area fraction from the two data sources))

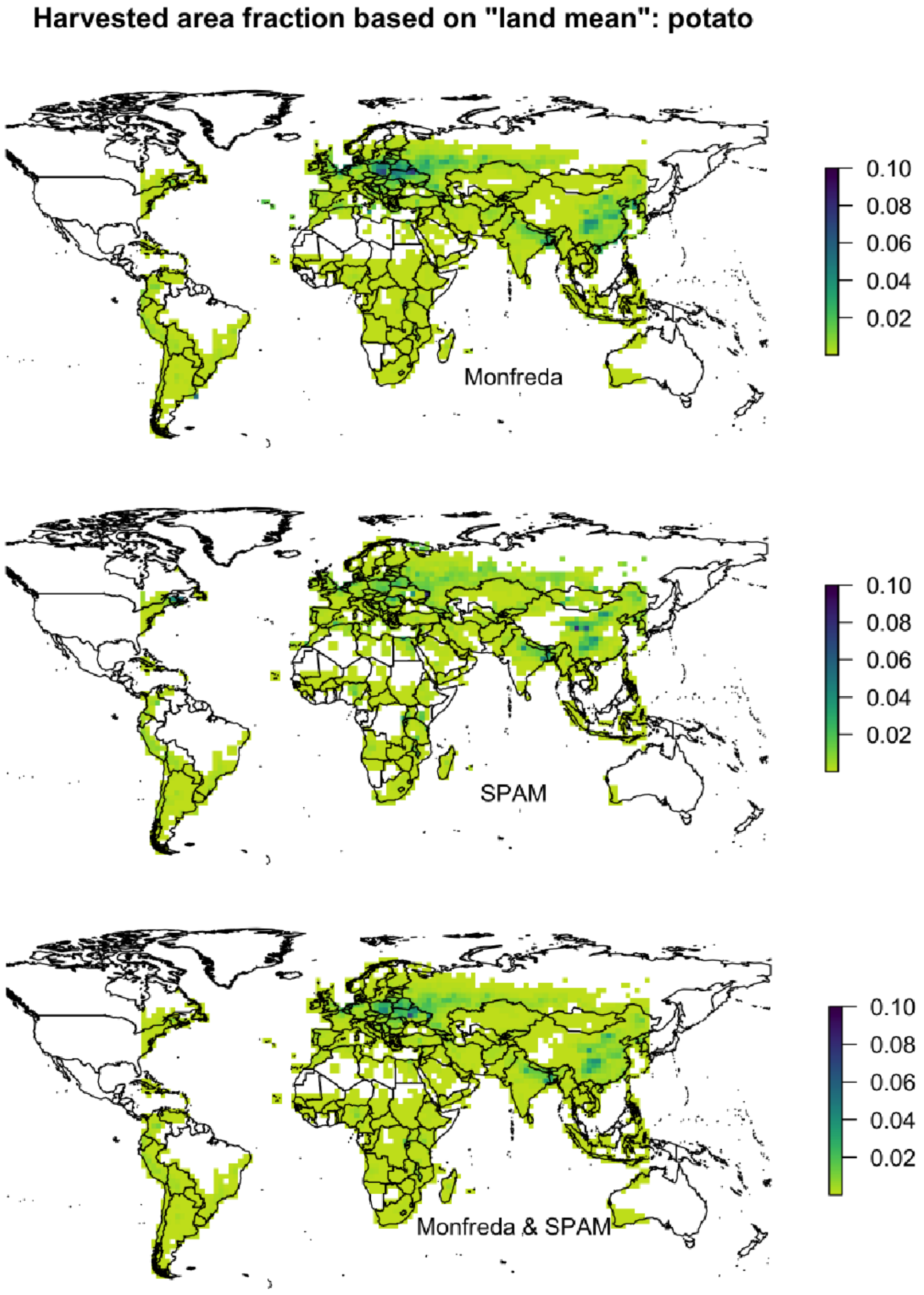

Figure S4a2. Maps of the harvested area fraction for **potato** based on the "**land mean**", the mean of all units within the area being aggregated that represent land (2-degree resolution, aggregated from 5-minute resolution in Monfreda et al. 2008, MapSPAM2005v3.2, and "Monfreda & SPAM" (the mean of the harvested area fraction from the two data sources))

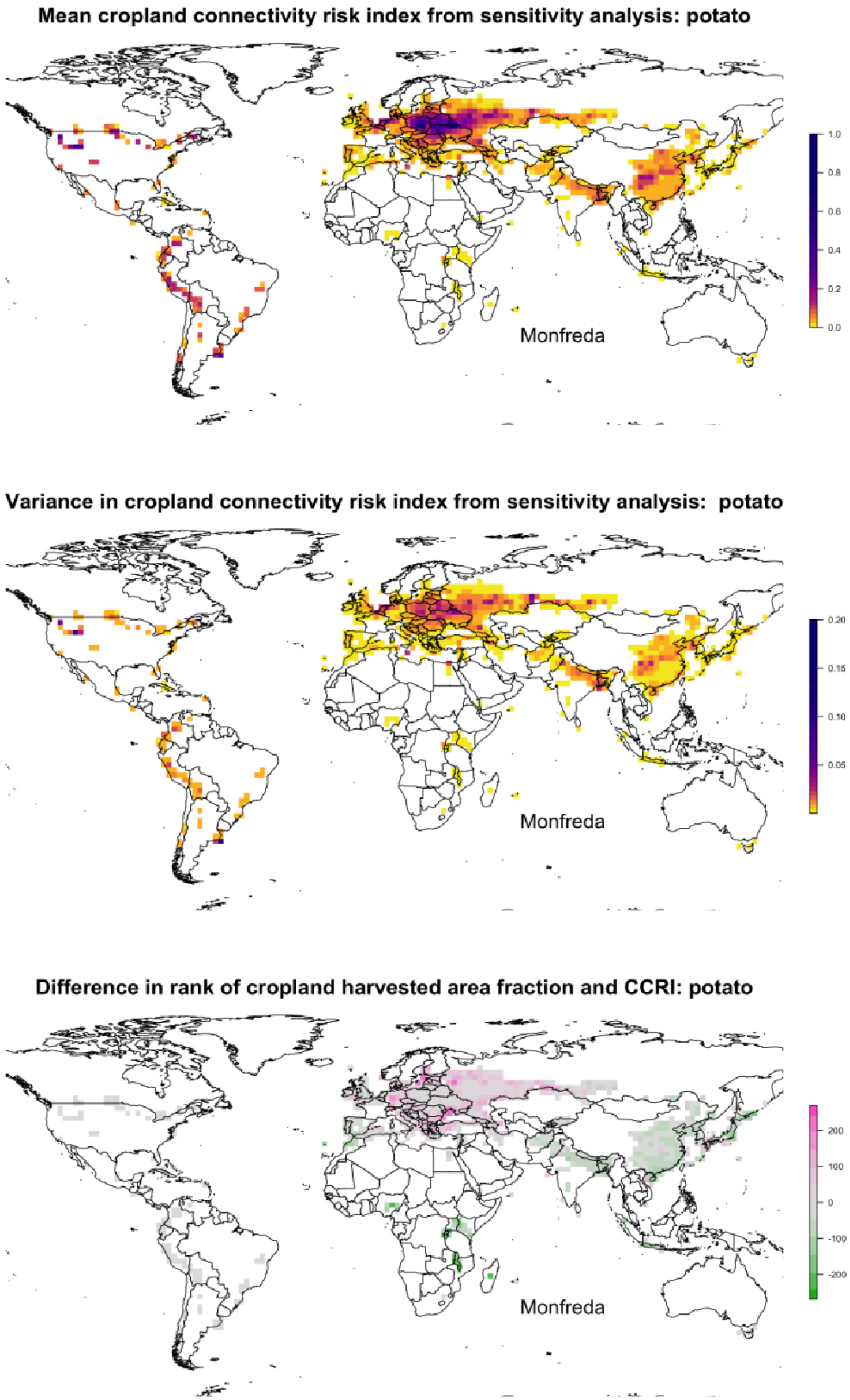

Figure S4b1. The uncertainty quantification analysis of the cropland connectivity risk index for **potato** using the Monfreda et al. 2008 data set

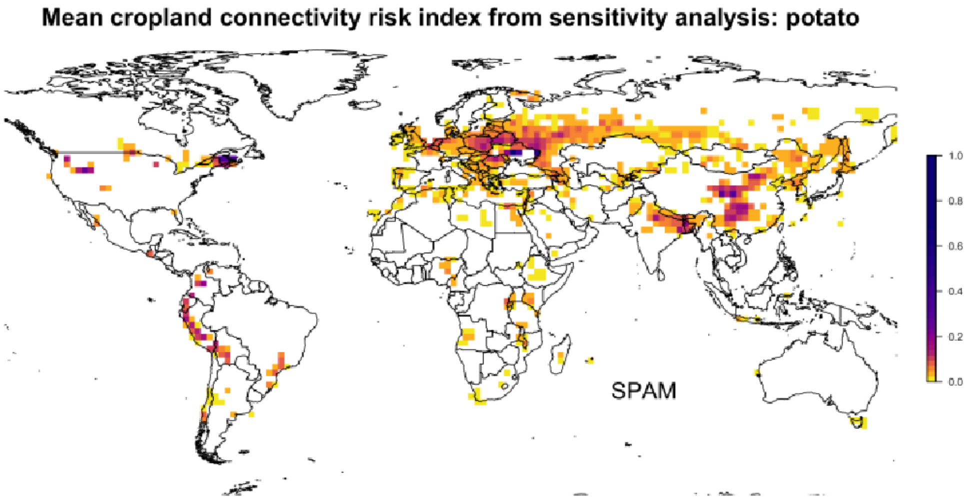

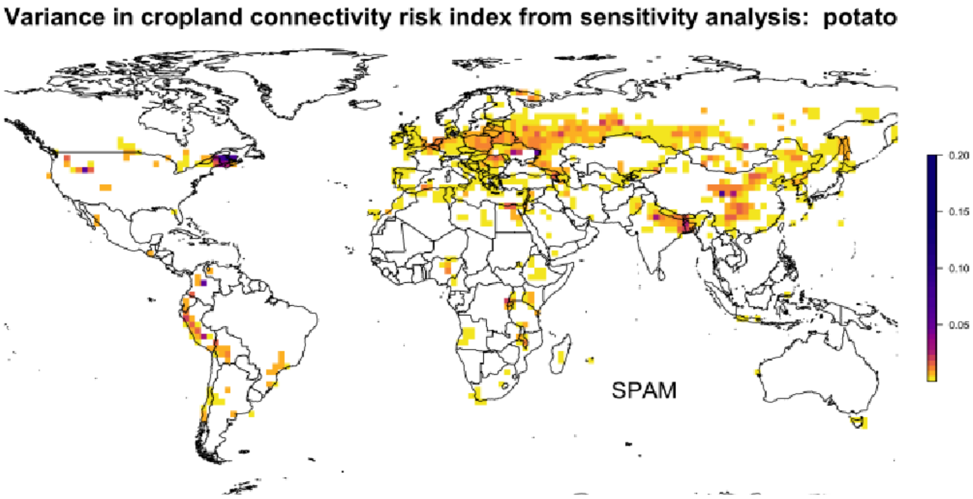

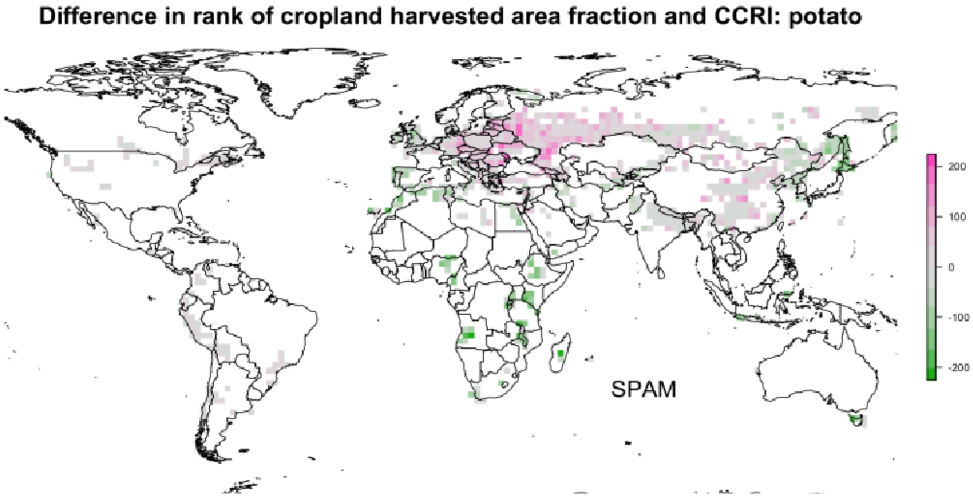

Figure S4b2. The uncertainty quantification analysis of the cropland connectivity risk index for **potato** using the MapSPAM2005v3.2 data set

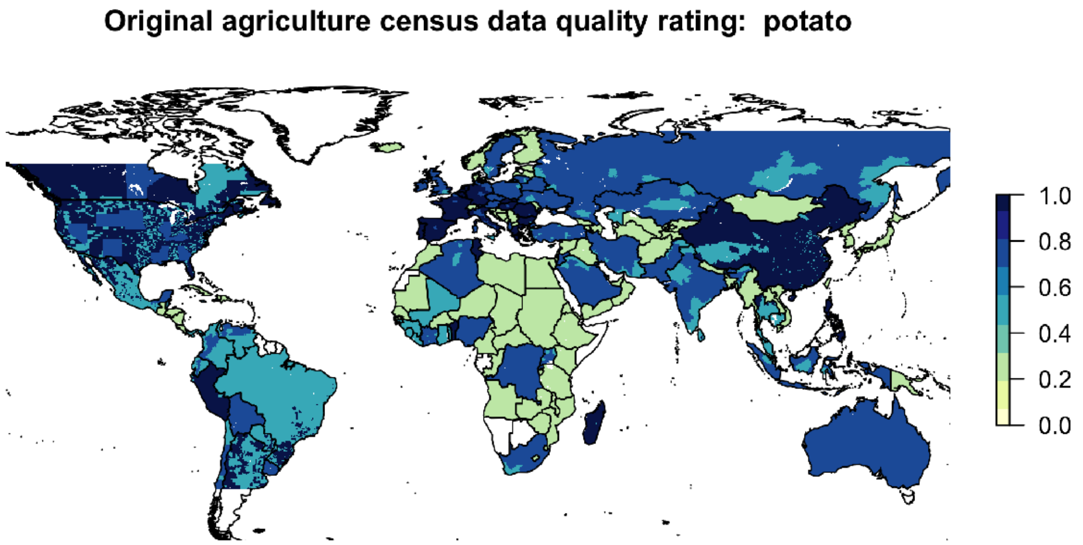

Figure S4c. Data quality map from Monfreda et al. 2008 for harvested area fraction for **potato**

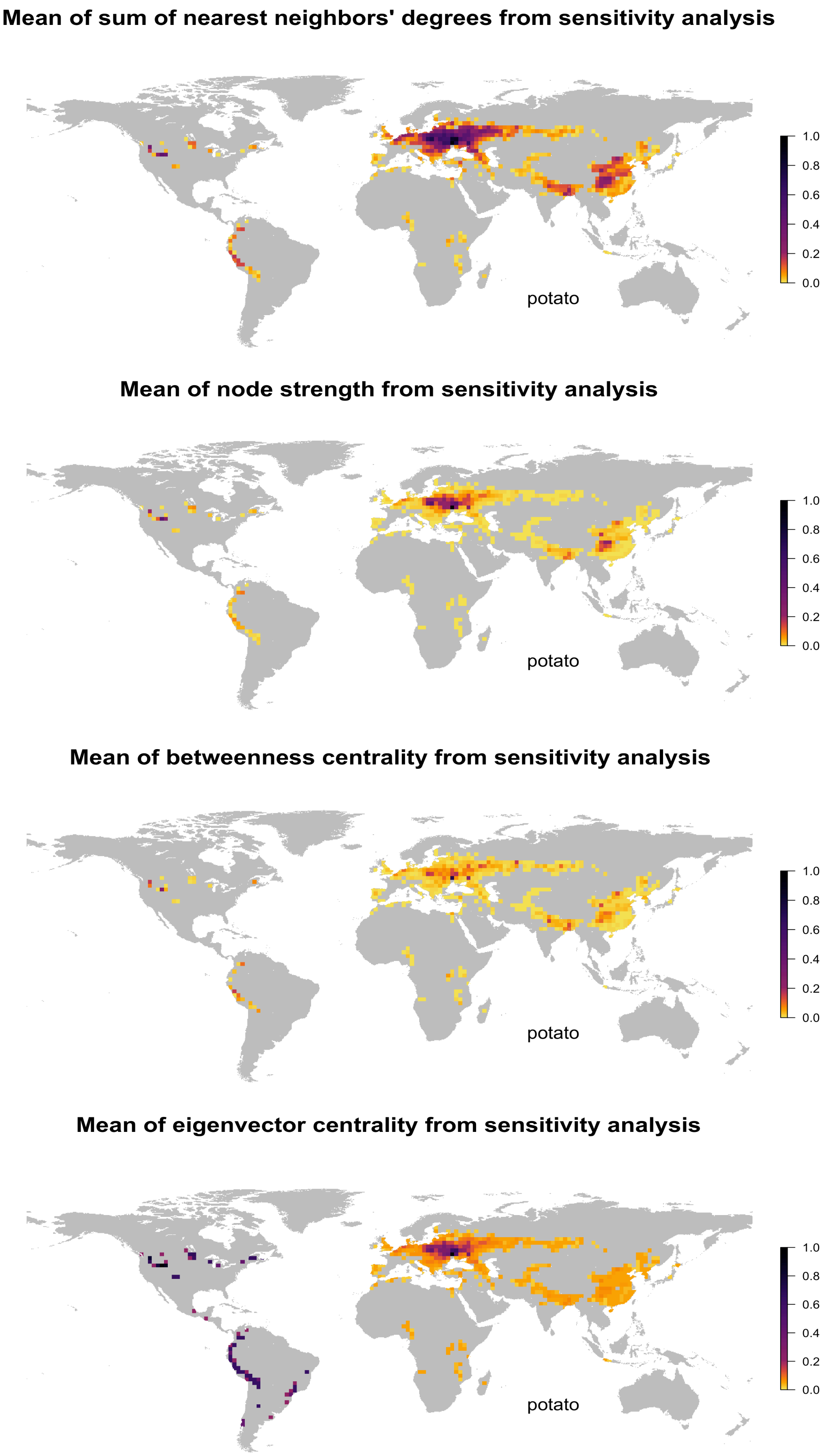

Figure S4d. The CCRI is the weighted mean of four measures of node centrality, with the mean from uncertainty quantification for each illustrated separately here for potato using "Monfreda & SPAM" (the mean of the harvested area fraction from the two data sources).
